# 2-(3-Fluoro­phen­yl)-3-methyl­sulfanyl-5-phenyl-1-benzofuran

**DOI:** 10.1107/S1600536811042541

**Published:** 2011-10-22

**Authors:** Pil Ja Seo, Hong Dae Choi, Byeng Wha Son, Uk Lee

**Affiliations:** a Department of Chemistry, Dongeui University, San 24 Kaya-dong Busanjin-gu, Busan 614-714, Republic of Korea; b Department of Chemistry, Pukyong National University, 599-1 Daeyeon 3-dong, Nam-gu, Busan 608-737, Republic of Korea

## Abstract

In the title compound, C_21_H_15_FOS, the dihedral angles between the mean plane of the benzofuran fragment and the pendant 3-fluoro­phenyl and phenyl rings are 1.76 (5) and 32.29 (5)°, respectively. In the crystal, mol­ecules are linked by a slipped π–π inter­action between the furan and benzene rings of neighbouring mol­ecules [centroid–centroid distance = 3.665 (2) Å, inter­planar distance = 3.391 (2) Å and slippage = 1.390 (2) Å].

## Related literature

For the pharmacological activity of benzofuran compounds, see: Aslam *et al.* (2009 ▶); Galal *et al.* (2009 ▶); Khan *et al.* (2005 ▶). For natural products with benzofuran rings, see: Akgul & Anil (2003 ▶); Soekamto *et al.* (2003 ▶). For related structures, see: Choi *et al.* (2009 ▶, 2010 ▶).
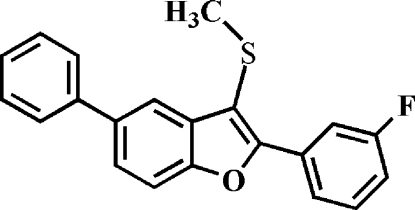


## Experimental

### 

#### Crystal data

C_21_H_15_FOS
*M* *_r_* = 334.39
Triclinic, 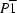

*a* = 4.7692 (1) Å
*b* = 9.6442 (2) Å
*c* = 17.4049 (3) Å
α = 89.7700 (1)°
β = 87.589 (1)°
γ = 80.061 (1)°
*V* = 787.82 (3) Å^3^
*Z* = 2
Mo *K*α radiation
μ = 0.22 mm^−1^
*T* = 296 K
0.34 × 0.25 × 0.12 mm

#### Data collection

Bruker SMART APEXII CCD diffractometer
Absorption correction: multi-scan (*SADABS*; Bruker, 2009 ▶) *T*_min_ = 0.929, *T*_max_ = 0.974
13895 measured reflections
3590 independent reflections
3234 reflections with *I* > 2σ(*I*)
*R*_int_ = 0.023

#### Refinement

*R*[*F* ^2^ > 2σ(*F* ^2^)] = 0.034
*wR*(*F* ^2^) = 0.091
*S* = 1.04
3590 reflections
218 parameters
H-atom parameters constrained
Δρ_max_ = 0.30 e Å^−3^
Δρ_min_ = −0.25 e Å^−3^

### 

Data collection: *APEX2* (Bruker, 2009 ▶); cell refinement: *SAINT* (Bruker, 2009 ▶); data reduction: *SAINT*; program(s) used to solve structure: *SHELXS97* (Sheldrick, 2008 ▶); program(s) used to refine structure: *SHELXL97* (Sheldrick, 2008 ▶); molecular graphics: *ORTEP-3* (Farrugia, 1997 ▶) and *DIAMOND* (Brandenburg, 1998 ▶); software used to prepare material for publication: *SHELXL97*.

## Supplementary Material

Crystal structure: contains datablock(s) global, I. DOI: 10.1107/S1600536811042541/ff2033sup1.cif

Structure factors: contains datablock(s) I. DOI: 10.1107/S1600536811042541/ff2033Isup2.hkl

Supplementary material file. DOI: 10.1107/S1600536811042541/ff2033Isup3.cml

Additional supplementary materials:  crystallographic information; 3D view; checkCIF report

## Figures and Tables

**Fig. 1.**
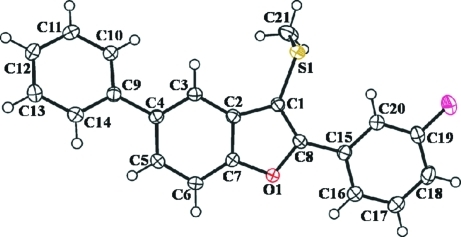
The molecular structure of the title compound with the atom numbering scheme. Displacement ellipsoids are drawn at the 50% probability level. H atoms are presented as small spheres of arbitrary radius.

**Fig. 2.**
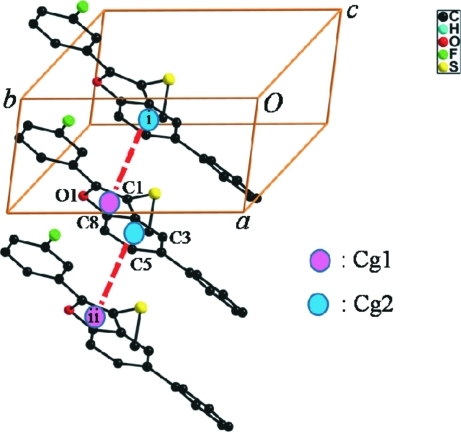
A view of the π–π interactions (dotted lines) in the crystal structure of the title compound. H atoms were omitted for clarity. [Symmetry codes: (i) x - 1, y, z; (ii) x + 1, y, z.]

## supplementary crystallographic information

### Comment

Recently, many compounds having a benzofuran moiety have drawn much attention due to their valuable pharmacological properties such as antibacterial and antifungal, antitumor and antiviral, and antimicrobial activities (Aslam *et al.*, 2009, Galal *et al.*, 2009, Khan *et al.*, 2005). These benzofuran derivatives occur in a wide range of natural products (Akgul & Anil, 2003; Soekamto *et al.*, 2003). As a part of our ongoing study of benzofuranderivatives containing either 2-(4-fluorophenyl) (Choi *et al.*, 2009) or 2-(4-chlorophenyl) (Choi *et al.*, 2010) substituents, we report herein the crystal structure of the title compound.

In the title molecule (Fig. 1), the benzofuran unit is essentially planar, with a mean deviation of 0.007 (1) Å from the least-squares plane defined by the nine constituent atoms. The dihedral angles between the mean plane of the benzofuran fragment and the 4-fluorophenyl and phenyl rings are 1.76 (5)° and 32.29 (5)°, respectively. The crystal packing (Fig. 2) is stabilized by a slipped π–π interaction between the furan and benzene rings of adjacent molecules, with a Cg1···Cg2^i^ distance of 3.665 (2) Å and an interplanar distance of 3.391 (2) Å resulting in a slippage of 1.390 (2) Å (Cg1 and Cg2 are the centroids of the C1/C2/C7/O1/C8 furan ring and the C2–C7 benzene ring, respectively).

### Experimental

Zinc chloride (273 mg, 2.0 mmol) was added to a stirred solution of 4-phenylphenol (340 mg, 2.0 mmol) and 2-chloro-2-methylsulfanyl-3'-fluoroacetophenone (437 mg, 2.0 mmol) in dichloromethane (30 mL) at room temperature, and stirring was continued at the same temperature for 1h. The reaction was quenched by the addition of water and the organic layer was separated, dried over magnesium sulfate, filtered and concentrated at reduced pressure. The residue was purified by column chromatography (hexane–benzene, 5:2 v/v) to afford the title compound as a colorless solid [yield 54%, m.p. 357–358 K; *R*_f_ = 0.61 (hexane–benzene, 5:2 v/v)]. Single crystals suitable for X-ray diffraction were prepared by slow evaporation of a solution of the title compound in acetone at room temperature.

### Refinement

All H atoms were positioned geometrically and refined using a riding model, with C—H = 0.95 Å for aryl and 0.98 Å for methyl H atoms. *U*_iso_(H) =1.2*U*_eq_(C) for aryl and 1.5*U*_eq_(C) for methyl H atoms.

### Crystal data

**Table:**

C_21_H_15_FOS	*Z* = 2
*M**_r_* = 334.39	*F*(000) = 348
Triclinic, *P*1	*D*_x_ = 1.410 Mg m^−^^3^
Hall symbol: -P 1	Mo *K*α radiation, λ = 0.71073 Å
*a* = 4.7692 (1) Å	Cell parameters from 7407 reflections
*b* = 9.6442 (2) Å	θ = 2.3–27.5°
*c* = 17.4049 (3) Å	µ = 0.22 mm^−^^1^
α = 89.7700 (1)°	*T* = 296 K
β = 87.589 (1)°	Block, colourless
γ = 80.061 (1)°	0.34 × 0.25 × 0.12 mm
*V* = 787.82 (3) Å^3^	

### Data collection

**Table:**

Bruker SMART APEXII CCD diffractometer	3590 independent reflections
Radiation source: rotating anode	3234 reflections with *I* > 2σ(*I*)
graphite multilayer	*R*_int_ = 0.023
Detector resolution: 10.0 pixels mm^-1^	θ_max_ = 27.5°, θ_min_ = 2.1°
φ and ω scans	*h* = −6→6
Absorption correction: multi-scan (*SADABS*; Bruker, 2009)	*k* = −12→12
*T*_min_ = 0.929, *T*_max_ = 0.974	*l* = −22→22
13895 measured reflections	

### Refinement

**Table:**

Refinement on *F*^2^	Primary atom site location: structure-invariant direct methods
Least-squares matrix: full	Secondary atom site location: difference Fourier map
*R*[*F*^2^ > 2σ(*F*^2^)] = 0.034	Hydrogen site location: difference Fourier map
*wR*(*F*^2^) = 0.091	H-atom parameters constrained
*S* = 1.04	*w* = 1/[σ^2^(*F*_o_^2^) + (0.0451*P*)^2^ + 0.3462*P*] where *P* = (*F*_o_^2^ + 2*F*_c_^2^)/3
3590 reflections	(Δ/σ)_max_ < 0.001
218 parameters	Δρ_max_ = 0.30 e Å^−^^3^
0 restraints	Δρ_min_ = −0.25 e Å^−^^3^

### Special details

**Table:**

Geometry. All esds (except the esd in the dihedral angle between two l.s. planes) are estimated using the full covariance matrix. The cell esds are taken into account individually in the estimation of esds in distances, angles and torsion angles; correlations between esds in cell parameters are only used when they are defined by crystal symmetry. An approximate (isotropic) treatment of cell esds is used for estimating esds involving l.s. planes.
Refinement. Refinement of F^2^ against ALL reflections. The weighted R-factor wR and goodness of fit S are based on F^2^, conventional R-factors R are based on F, with F set to zero for negative F^2^. The threshold expression of F^2^ > 2sigma(F^2^) is used only for calculating R-factors(gt) etc. and is not relevant to the choice of reflections for refinement. R-factors based on F^2^ are statistically about twice as large as those based on F, and R- factors based on ALL data will be even larger.

### Fractional atomic coordinates and isotropic or equivalent isotropic displacement parameters (Å^2^)

**Table:**

	*x*	*y*	*z*	*U*_iso_*/*U*_eq_	
S1	0.94952 (7)	0.43069 (3)	0.147672 (18)	0.02311 (10)	
F1	0.2102 (2)	0.79398 (10)	0.01953 (5)	0.0391 (2)	
O1	1.05781 (19)	0.76869 (9)	0.26561 (5)	0.0218 (2)	
C1	1.0423 (3)	0.56199 (13)	0.20611 (7)	0.0196 (2)	
C2	1.2552 (3)	0.53713 (13)	0.26345 (7)	0.0199 (2)	
C3	1.4383 (3)	0.41893 (13)	0.28916 (7)	0.0198 (2)	
H3	1.4391	0.3310	0.2674	0.024*	
C4	1.6195 (3)	0.43454 (13)	0.34781 (7)	0.0200 (2)	
C5	1.6144 (3)	0.56915 (14)	0.38003 (7)	0.0232 (3)	
H5	1.7378	0.5787	0.4189	0.028*	
C6	1.4325 (3)	0.68749 (14)	0.35590 (7)	0.0238 (3)	
H6	1.4295	0.7757	0.3777	0.029*	
C7	1.2556 (3)	0.66699 (13)	0.29759 (7)	0.0205 (2)	
C8	0.9298 (3)	0.70255 (13)	0.20980 (7)	0.0203 (2)	
C9	1.8215 (3)	0.31190 (13)	0.37610 (7)	0.0211 (3)	
C10	1.9415 (3)	0.20260 (14)	0.32642 (8)	0.0248 (3)	
H10	1.8910	0.2055	0.2753	0.030*	
C11	2.1353 (3)	0.08960 (14)	0.35218 (8)	0.0308 (3)	
H11	2.2143	0.0176	0.3184	0.037*	
C12	2.2111 (4)	0.08401 (15)	0.42809 (9)	0.0367 (4)	
H12	2.3420	0.0087	0.4453	0.044*	
C13	2.0914 (4)	0.19081 (16)	0.47824 (9)	0.0362 (4)	
H13	2.1406	0.1867	0.5294	0.043*	
C14	1.8985 (3)	0.30393 (15)	0.45263 (8)	0.0279 (3)	
H14	1.8195	0.3753	0.4868	0.034*	
C15	0.7126 (3)	0.79758 (13)	0.16924 (7)	0.0212 (3)	
C16	0.6597 (3)	0.94127 (15)	0.18697 (8)	0.0299 (3)	
H16	0.7610	0.9756	0.2250	0.036*	
C17	0.4572 (3)	1.03289 (16)	0.14822 (9)	0.0357 (3)	
H17	0.4239	1.1281	0.1608	0.043*	
C18	0.3041 (3)	0.98512 (16)	0.09120 (8)	0.0315 (3)	
H18	0.1692	1.0464	0.0648	0.038*	
C19	0.3592 (3)	0.84369 (15)	0.07508 (8)	0.0262 (3)	
C20	0.5568 (3)	0.74876 (14)	0.11223 (7)	0.0237 (3)	
H20	0.5859	0.6536	0.0995	0.028*	
C21	1.2261 (3)	0.41386 (17)	0.07312 (9)	0.0352 (3)	
H21A	1.2159	0.5014	0.0461	0.053*	
H21B	1.2017	0.3410	0.0379	0.053*	
H21C	1.4086	0.3902	0.0956	0.053*	

### Atomic displacement parameters (Å^2^)

**Table:**

	*U*^11^	*U*^22^	*U*^33^	*U*^12^	*U*^13^	*U*^23^
S1	0.02386 (17)	0.02130 (17)	0.02495 (17)	−0.00512 (12)	−0.00487 (12)	−0.00323 (12)
F1	0.0392 (5)	0.0395 (5)	0.0387 (5)	−0.0029 (4)	−0.0205 (4)	−0.0007 (4)
O1	0.0230 (5)	0.0190 (4)	0.0229 (4)	−0.0008 (3)	−0.0057 (3)	−0.0021 (3)
C1	0.0188 (6)	0.0206 (6)	0.0196 (6)	−0.0038 (4)	−0.0018 (4)	−0.0009 (4)
C2	0.0189 (6)	0.0217 (6)	0.0194 (6)	−0.0048 (5)	−0.0001 (4)	−0.0014 (5)
C3	0.0204 (6)	0.0181 (6)	0.0212 (6)	−0.0042 (4)	−0.0001 (4)	−0.0014 (4)
C4	0.0204 (6)	0.0192 (6)	0.0204 (6)	−0.0033 (5)	−0.0001 (4)	0.0004 (4)
C5	0.0248 (6)	0.0236 (6)	0.0214 (6)	−0.0035 (5)	−0.0054 (5)	−0.0022 (5)
C6	0.0278 (7)	0.0193 (6)	0.0243 (6)	−0.0029 (5)	−0.0040 (5)	−0.0048 (5)
C7	0.0204 (6)	0.0186 (6)	0.0217 (6)	−0.0009 (4)	−0.0021 (5)	0.0003 (5)
C8	0.0204 (6)	0.0222 (6)	0.0189 (6)	−0.0051 (5)	−0.0018 (4)	−0.0016 (5)
C9	0.0208 (6)	0.0198 (6)	0.0235 (6)	−0.0053 (5)	−0.0033 (5)	0.0022 (5)
C10	0.0297 (7)	0.0203 (6)	0.0245 (6)	−0.0038 (5)	−0.0056 (5)	0.0008 (5)
C11	0.0365 (8)	0.0198 (6)	0.0342 (8)	0.0015 (5)	−0.0069 (6)	−0.0032 (5)
C12	0.0435 (9)	0.0221 (7)	0.0427 (9)	0.0034 (6)	−0.0199 (7)	0.0010 (6)
C13	0.0486 (9)	0.0287 (7)	0.0307 (7)	−0.0009 (6)	−0.0183 (7)	0.0010 (6)
C14	0.0344 (7)	0.0241 (7)	0.0245 (7)	−0.0015 (5)	−0.0061 (5)	−0.0022 (5)
C15	0.0189 (6)	0.0223 (6)	0.0219 (6)	−0.0024 (5)	0.0002 (5)	0.0012 (5)
C16	0.0318 (7)	0.0243 (7)	0.0328 (7)	−0.0010 (5)	−0.0093 (6)	−0.0027 (5)
C17	0.0408 (8)	0.0227 (7)	0.0413 (8)	0.0030 (6)	−0.0113 (7)	−0.0011 (6)
C18	0.0284 (7)	0.0310 (7)	0.0327 (7)	0.0030 (6)	−0.0067 (6)	0.0060 (6)
C19	0.0229 (6)	0.0330 (7)	0.0233 (6)	−0.0054 (5)	−0.0049 (5)	0.0007 (5)
C20	0.0235 (6)	0.0235 (6)	0.0240 (6)	−0.0031 (5)	−0.0023 (5)	0.0000 (5)
C21	0.0333 (8)	0.0419 (9)	0.0319 (7)	−0.0110 (6)	0.0015 (6)	−0.0137 (6)

### Geometric parameters (Å, °)

**Table:**

S1—C1	1.7537 (13)	C10—H10	0.9300
S1—C21	1.7984 (15)	C11—C12	1.383 (2)
F1—C19	1.3597 (16)	C11—H11	0.9300
O1—C7	1.3719 (14)	C12—C13	1.382 (2)
O1—C8	1.3830 (15)	C12—H12	0.9300
C1—C8	1.3698 (17)	C13—C14	1.3854 (19)
C1—C2	1.4435 (17)	C13—H13	0.9300
C2—C7	1.3887 (17)	C14—H14	0.9300
C2—C3	1.3955 (17)	C15—C20	1.3946 (18)
C3—C4	1.3897 (17)	C15—C16	1.3984 (19)
C3—H3	0.9300	C16—C17	1.387 (2)
C4—C5	1.4122 (18)	C16—H16	0.9300
C4—C9	1.4873 (17)	C17—C18	1.383 (2)
C5—C6	1.3843 (18)	C17—H17	0.9300
C5—H5	0.9300	C18—C19	1.372 (2)
C6—C7	1.3829 (18)	C18—H18	0.9300
C6—H6	0.9300	C19—C20	1.3758 (18)
C8—C15	1.4629 (17)	C20—H20	0.9300
C9—C10	1.3942 (18)	C21—H21A	0.9600
C9—C14	1.3948 (18)	C21—H21B	0.9600
C10—C11	1.3879 (18)	C21—H21C	0.9600
			
C1—S1—C21	101.72 (6)	C10—C11—H11	120.0
C7—O1—C8	106.60 (9)	C13—C12—C11	119.73 (13)
C8—C1—C2	106.46 (11)	C13—C12—H12	120.1
C8—C1—S1	129.09 (10)	C11—C12—H12	120.1
C2—C1—S1	124.41 (9)	C12—C13—C14	120.36 (13)
C7—C2—C3	119.37 (11)	C12—C13—H13	119.8
C7—C2—C1	105.75 (11)	C14—C13—H13	119.8
C3—C2—C1	134.88 (12)	C13—C14—C9	120.69 (13)
C4—C3—C2	119.05 (11)	C13—C14—H14	119.7
C4—C3—H3	120.5	C9—C14—H14	119.7
C2—C3—H3	120.5	C20—C15—C16	118.60 (12)
C3—C4—C5	119.45 (12)	C20—C15—C8	121.58 (12)
C3—C4—C9	120.87 (11)	C16—C15—C8	119.82 (12)
C5—C4—C9	119.67 (11)	C17—C16—C15	120.44 (13)
C6—C5—C4	122.43 (12)	C17—C16—H16	119.8
C6—C5—H5	118.8	C15—C16—H16	119.8
C4—C5—H5	118.8	C18—C17—C16	121.14 (14)
C7—C6—C5	116.14 (12)	C18—C17—H17	119.4
C7—C6—H6	121.9	C16—C17—H17	119.4
C5—C6—H6	121.9	C19—C18—C17	117.29 (13)
O1—C7—C6	125.91 (11)	C19—C18—H18	121.4
O1—C7—C2	110.53 (11)	C17—C18—H18	121.4
C6—C7—C2	123.56 (12)	F1—C19—C18	118.41 (12)
C1—C8—O1	110.66 (11)	F1—C19—C20	117.96 (13)
C1—C8—C15	135.74 (12)	C18—C19—C20	123.63 (13)
O1—C8—C15	113.59 (11)	C19—C20—C15	118.90 (12)
C10—C9—C14	118.30 (12)	C19—C20—H20	120.6
C10—C9—C4	120.71 (11)	C15—C20—H20	120.6
C14—C9—C4	120.98 (12)	S1—C21—H21A	109.5
C11—C10—C9	120.88 (12)	S1—C21—H21B	109.5
C11—C10—H10	119.6	H21A—C21—H21B	109.5
C9—C10—H10	119.6	S1—C21—H21C	109.5
C12—C11—C10	120.04 (13)	H21A—C21—H21C	109.5
C12—C11—H11	120.0	H21B—C21—H21C	109.5
			
C21—S1—C1—C8	100.68 (13)	C3—C4—C9—C10	32.27 (18)
C21—S1—C1—C2	−81.81 (12)	C5—C4—C9—C10	−146.70 (13)
C8—C1—C2—C7	−0.32 (13)	C3—C4—C9—C14	−148.48 (13)
S1—C1—C2—C7	−178.30 (9)	C5—C4—C9—C14	32.55 (18)
C8—C1—C2—C3	178.54 (13)	C14—C9—C10—C11	−0.8 (2)
S1—C1—C2—C3	0.6 (2)	C4—C9—C10—C11	178.47 (13)
C7—C2—C3—C4	−0.78 (18)	C9—C10—C11—C12	0.3 (2)
C1—C2—C3—C4	−179.52 (13)	C10—C11—C12—C13	0.5 (2)
C2—C3—C4—C5	0.00 (18)	C11—C12—C13—C14	−0.7 (3)
C2—C3—C4—C9	−178.97 (11)	C12—C13—C14—C9	0.1 (2)
C3—C4—C5—C6	0.65 (19)	C10—C9—C14—C13	0.6 (2)
C9—C4—C5—C6	179.63 (12)	C4—C9—C14—C13	−178.66 (14)
C4—C5—C6—C7	−0.48 (19)	C1—C8—C15—C20	2.0 (2)
C8—O1—C7—C6	−179.95 (12)	O1—C8—C15—C20	−178.56 (11)
C8—O1—C7—C2	−0.27 (13)	C1—C8—C15—C16	−177.34 (14)
C5—C6—C7—O1	179.30 (12)	O1—C8—C15—C16	2.05 (17)
C5—C6—C7—C2	−0.4 (2)	C20—C15—C16—C17	−0.3 (2)
C3—C2—C7—O1	−178.71 (10)	C8—C15—C16—C17	179.10 (13)
C1—C2—C7—O1	0.36 (14)	C15—C16—C17—C18	−0.3 (2)
C3—C2—C7—C6	0.99 (19)	C16—C17—C18—C19	0.5 (2)
C1—C2—C7—C6	−179.94 (12)	C17—C18—C19—F1	179.64 (13)
C2—C1—C8—O1	0.17 (14)	C17—C18—C19—C20	−0.1 (2)
S1—C1—C8—O1	178.03 (9)	F1—C19—C20—C15	179.80 (11)
C2—C1—C8—C15	179.58 (14)	C18—C19—C20—C15	−0.4 (2)
S1—C1—C8—C15	−2.6 (2)	C16—C15—C20—C19	0.63 (19)
C7—O1—C8—C1	0.05 (13)	C8—C15—C20—C19	−178.76 (12)
C7—O1—C8—C15	−179.50 (10)		

## References

[bb1] Akgul, Y. Y. & Anil, H. (2003). *Phytochemistry*, **63**, 939–943. 10.1016/s0031-9422(03)00357-1 12895543

[bb2] Aslam, S. N., Stevenson, P. C., Kokubun, T. & Hall, D. R. (2009). *Microbiol. Res.* 164, 191-195. 10.1016/j.micres.2006.11.012 17418552

[bb3] Brandenburg, K. (1998). *DIAMOND* Crystal Impact GbR, Bonn, Germany.

[bb4] Bruker (2009). *APEX2*, *SADABS* and *SAINT* Bruker AXS Inc., Madison, Wisconsin, USA.

[bb5] Choi, H. D., Seo, P. J., Son, B. W. & Lee, U. (2009). *Acta Cryst.* E**65**, o2766. 10.1107/S1600536809041713 PMC2971277 21578360

[bb6] Choi, H. D., Seo, P. J., Son, B. W. & Lee, U. (2010). *Acta Cryst.* E**66**, o802. 10.1107/S1600536810008706 PMC2983985 21580638

[bb7] Farrugia, L. J. (1997). *J. Appl. Cryst.* **30**, 565.

[bb8] Galal, S. A., Abd El-All, A. S., Abdallah, M. M. & El-Diwani, H. I. (2009). *Bioorg. Med. Chem. Lett* **19**, 2420-2428. 10.1016/j.bmcl.2009.03.069 19345581

[bb9] Khan, M. W., Alam, M. J., Rashid, M. A. & Chowdhury, R. (2005). *Bioorg. Med. Chem* **13**, 4796–4805. 10.1016/j.bmc.2005.05.009 15964760

[bb10] Sheldrick, G. M. (2008). *Acta Cryst.* A**64**, 112–122. 10.1107/S0108767307043930 18156677

[bb11] Soekamto, N. H., Achmad, S. A., Ghisalberti, E. L., Hakim, E. H. & Syah, Y. M. (2003). *Phytochemistry*, **64**, 831–834. 10.1016/j.phytochem.2003.08.009 14559276

